# The complete mitochondrial genome of *Cynoglossus joyneri* and its novel rearrangement

**DOI:** 10.1080/23802359.2017.1365642

**Published:** 2017-09-01

**Authors:** Zhang Bo, Jia Lei, Liu Kefeng, Sun Jinsheng

**Affiliations:** Tianjin Bohai Sea Fisheries Research Institute, Tianjin, China

**Keywords:** *Cynoglossus joyneri*, rearrangement, translocation

## Abstract

The complete mitochondrial genome of *Cynoglossus joyneri* was determined. The length of the *C. joyneri* complete mitochondrial DNA sequence is 16,428 bp. The tRNA Gln gene is translocated from the light to the heavy strand (Q inversion), which is accompanied by shuffling of the tRNA Ile gene and long-range translocation of the putative control region downstream to a site between ND1 and the tRNA Gln gene, which is also seen in the closely related *Cynoglossus semilaevis*. *C. joyneri* has a closer relationship to *Cynoglossus lineolatus* and *C. semilaevis*, but had a distant relationship to *Phoxinus percnurus* and *Sternula albifrons.*

Here, we report a novel rearrangement in the mitogenome of *Cynoglossus joyneri* Günther. We made a striking discovery that the tRNA Gln gene has been translocated from the light to the heavy strand (Q inversion), and this has been accompanied by shuffling of the tRNA Ile gene and long-range translocation of the putative control region downstream to a site between ND1 and the tRNA Gln gene.

*Cynoglossus joyneri* samples were collected from Bohai sea (Tianjin, China), and the geospatial coordinates are 38°07′35″N and 118°10′16″E. All specimens were preserved in 100% ethanol and total genomic DNA was obtained by phenol chloroform extraction from the fin tissue of *C. joyneri*.

Twelve pairs of PCR primers were designed based on the mtDNA sequences of *Cynoglossus semilaevis*. The PCR products were firstly detected by visualization in a 1% agarose gel electrophoresis, and then sequenced by using DNA Sequencer at BGI Inc. Phylogenetic analyses were performed using the Neighbour-Joining (NJ) method and Maximum Likelihood (ML).

The length of the complete mitochondrial DNA sequence was 16,428 bp, consisting of 13 protein-coding genes, 22 tRNA genes, and two rRNA genes (GenBank accession number: KU754054). The 12S and 16S rRNA genes in *C. joyneri* mtDNA were 939 bp and 1676 bp, respectively. In contrast to *C. semilaevis*, our analysis showed that the 16S rRNA has a higher A + T content (60.7%) than the 12S rRNA (55.7%). The size and position of the 13 protein-coding genes in *C. joyneri* mtDNA are consistent with those of other bony fish. The ND3 gene sequence started with ATA, and CO1 coding sequence started with GTG, similar to other *Cynoglossus* species. Phylogenetic trees were constructed using the NJ methods (Figure 1). Throughout the phylogenetic analysis, *C. joyneri* had a closer relationship to *C. lineolatus* and *C. semilaevis*.

**Figure 1.**
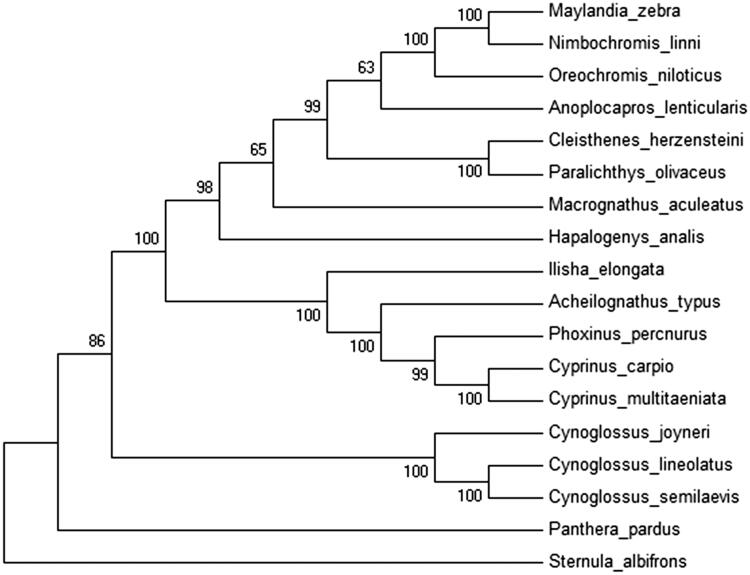
The consensus phylogenetic relationship of *C. joyneri* with the other species from Neighbour-Joining (NJ) analyses. The numbers on the branches are bootstrap values for NJ.

In *C. joyneri*, the tRNA Gln gene has been translocated from the light to the heavy strand (Q inversion). This is accompanied by shuffling of the tRNA Ile gene and long-range translocation of the putative control region downstream to a site between the ND1 and tRNA Gln genes. The new gene order may be a synapomorphic character of this group.

